# Benefits and Safety of Chinese Herbal Medicine in Treating Psoriasis: An Overview of Systematic Reviews

**DOI:** 10.3389/fphar.2021.680172

**Published:** 2021-07-01

**Authors:** Jie Zhang, Qianying Yu, Li Peng, Yuesi Qin, Mingyi Jing, Dan Huang, Jing Guo, Min Xiao, Mingling Chen

**Affiliations:** ^1^ Hospital of Chengdu University of Traditional Chinese Medicine, Chengdu, China; ^2^ Chengdu University of Traditional Chinese Medicine, Chengdu, China; ^3^ Chengdu Integrated TCM and Western Medicine Hospital, Chengdu, China

**Keywords:** Chinese herbal medicine, psoriasis, systematic review, AMSTAR-2, ROBIS, PRISMA, grade

## Abstract

**Background:** In recent years, systematic reviews/meta-analyses (SRs/MAs) of Chinese herbal medicine (CHM) for psoriasis have continuously emerged. Their methods and evidence quality, however, are yet to be evaluated, and whether their conclusions can provide clinicians with reliable evidence is still debatable.

**Objectives:** This overview aims to evaluate the methodological quality, risk of bias, and reporting quality of relevant SRs/MAs, as well as the current evidence of CHM for treating psoriasis.

**Methods:** We searched nine electronic databases from their respective time of establishment to January 20, 2021, as well as the reference lists of the included SRs/MAs, protocol registries, and gray literature. Two reviewers independently used the following: A Measurement Tool to Assess Systematic Reviews (AMSTAR) 2, Risk of Bias in Systematic Reviews (ROBIS), the Preferred Reporting Items for Systematic Reviews and Meta-analyses (PRISMA), and Grades of Recommendations, Assessment, Development and Evaluation (GRADE) to evaluate the methodological quality, risk of bias, reporting quality, and evidence quality of the included SRs/MAs.

**Results:** This review included 14 SRs/MAs involving 45 outcomes, of which 12 (85.71%) SRs/MAs had a very low quality evaluated by AMSTAR 2 and 7 (50.00%) SRs/MAs had a high risk of bias assessed by ROBIS. The protocol and registration and funding statements were the major reporting flaws according to the PRISMA checklist. The evaluation with the GRADE system demonstrated no outcome of high-quality evidence, and inconsistent efficacy evaluations were found in this overview. Only 15 (33.33%) outcomes were moderate-quality evidence, supporting the claim that CHM plus Western medicine (WM) was superior to WM. Generally low quality of evidence showed no difference in the incidence of adverse events between the combined therapy and WM. However, the conclusion that CHM was superior to WM cannot be drawn due to the inconsistent results.

**Conclusion:** Despite that CHM has the potential benefit and safety in the adjuvant treatment of psoriasis, the conclusion should be treated with caution because of the generally low quality of methodology and evidence. In the future, high-quality randomized controlled trials (RCTs) should be carried out, and the quality of relevant SRs should also be improved to promote their clinical application.

## Introduction

Psoriasis is a common chronic inflammatory dermatological disease. The typical lesions are scaly erythema or plaques that can be localized or widely distributed. In addition to skin lesions, patients with psoriasis present with joint symptoms, and those with moderate-to-severe psoriasis often have hypertension, Crohn’s disease, cancer, metabolic syndrome, depression, and other comorbidities, all of which seriously affect their quality of life. These comorbidities have been linked to an increase in the incidence and mortality of patients with psoriasis (Boehncke and Schön, 2015; von Csiky-Sessoms and Lebwohl, 2019). In recent years, epidemiological studies have shown that the prevalence and incidence of psoriasis are on the rise, with new cases increasing from 92 per 100,000 in 1990 to 99 per 100,000 in 2017. The highest incidence is found in North America and Western Europe, whereas the lowest is found in Asia (AlQassimi et al., 2020). The etiology and pathogenesis of psoriasis are not completely clear, and the current treatment protocol of Western medicine (WM) mainly includes topical therapy, phototherapy, conventional systemic therapy, and biologics (van der Kraaij et al., 2019). A standard treatment can effectively alleviate symptoms and even achieve a clinical cure. However, psoriasis is a chronic inflammatory dermatological disease that is prone to relapse, currently with no effective treatment to completely cure it (Association, 2019). Owing to various adverse reactions from long-term systemic therapy, high treatment costs, and difficulties associated with biological target identification, patients may have poor treatment satisfaction and low compliance (Nast et al., 2012; Armstrong et al., 2013; Tveit et al., 2019). Consequently, the number of patients with psoriasis who opt for complementary and alternative medicine for treatment is growing (Gamret et al., 2018).

Chinese herbal medicine (CHM), an indispensable part of complementary and alternative medicine, has a long history of use in the treatment of psoriasis. CHM is based on the principles of disease–syndrome combination, syndrome differentiation, holistic treatment, and treatment based on the stage of the disease, which can provide individualized treatment for patients with psoriasis. Through the multitarget and multipathway mechanism of action in the treatment of psoriasis, CHM has several therapeutic advantages that cannot be ignored or replaced by other therapies (Wang et al., 2020). The increasing evidence based on the SRs/MAs of CHM for treating psoriasis has suggested that CHM can improve clinical symptoms, decrease the psoriasis area and severity index (PASI) score, reduce the side effects of WM, delay the recurrence of psoriasis, and enhance the quality of life (Yang et al., 2015; Zhang et al., 2016; Guan et al., 2017; Xu et al., 2019). However, their qualities of methodology and evidence have not yet been assessed, and whether their conclusions can provide clinicians with credible evidence is still controversial. Therefore, this overview is conducted to assess the methodological quality, risk of bias, and reporting quality of SRs/MAs and to summarize and evaluate the current evidence of CHM for psoriasis. This overview aims to provide a reference for clinicians and to guide future high-quality RCTs and relevant SRs.

## Methods

The Preferred Reporting Items for Overviews of Systematic Reviews including harms checklist (Bougioukas et al., 2018) was used to perform this review. The overview protocol was registered with Open Science Framework (registration No.: DOI 10.17605/OSF.IO/VC654) and published in *Medicine* (Zhang et al., 2020).

### Eligibility Criteria

#### Type of Studies

The SRs/MAs based on randomized controlled trials (RCTs) of CHM for treating psoriasis were included. Overviews, narrative reviews, SR/MA protocols, comments, and conference abstracts with insufficient data were excluded. Furthermore, a network meta-analysis comparing the efficacy of different CHMs was also excluded.

#### Type of Participants

SRs/MAs with participants who had been diagnosed with psoriasis with either international or Chinese criteria were included. There were no restrictions for the recruitment of participants based on gender, age, ethnicity, duration, or stage of the disease. Studies with patients presenting with psoriatic arthritis or other comorbidities (such as hypertension, diabetes, and cancer) were excluded.

#### Type of Interventions

The experimental groups received treatment with either systemic use or topical applications of CHM alone or in combination with the active therapies (such as Western medicine, phototherapy, or other nonpharmacological therapies) with no restriction on dosage form, whereas the control groups received neither medications, placebos, nor the active therapies. Studies in which the experimental groups received acupuncture, massage, Tai Ji, or qigong were excluded in addition to those in which the control groups received treatment with CHM.

#### Type of Outcomes

SRs/MAs that considered the primary outcome as the total effective rate based on the decline rate of PASI score were included in our overview. Additionally, SRs/MAs that considered the secondary outcomes as the mean improvement in PASI score, quality of life score, itching score, traditional Chinese medicine syndrome score, recurrence rate, and adverse reactions were included.

### Search Strategy

Nine electronic databases were searched from the time of their respective establishments to January 20, 2021, including Medline (via OVID), Embase, Cochrane Library, AMED (via EBSCO), CINAHL (via EBSCO), Chinese Biological Medicine (CBM), China National Knowledge Infrastructure (CNKI), VIP, and Wan fang database. There were no restrictions on the language of the publication. The literature search applied MeSH terms or keywords combined with free-text words, such as psoriasis, traditional Chinese medicine, Chinese herbal drugs, systematic review, and meta-analysis, which were modified to suit different databases. Details of the search strategy for each database are presented in Supplementary Material 1. To prevent any SRs/MAs from being overlooked, we also screened the reference list of included SRs/MAs, protocol registries, and gray literature.

### Study Selection and Data Extraction

Reference management software (Endnote X9, Clavirate Analytics, United States) was used to manage the retrieved articles and to remove the duplicates. Based on the inclusion and exclusion criteria, two reviewers (JZ and QY) independently screened the titles and abstracts to find potential SRs/MAs. The full-text articles were then obtained for further screening to identify the eligibility. Author, publication year, sample size, intervention, outcomes, quality assessment methods, results, and conclusion were all extracted by two reviewers independently using a predefined data collection table. Finally, the extracted data were cross checked by two reviewers (LP and YQ) to eliminate the discrepancy.

### Quality Assessment of SRs/MAs

Two reviewers (JZ and QY) who had undergone training for evidence-based medicine independently performed and cross checked the quality assessment of the included SRs/MAs. Disagreements between the two reviewers were resolved by discussion and consensus or by consultation with a third reviewer (LP).

#### Assessment of Methodological Quality

AMSTAR 2 (Shea et al., 2017), a 16-item tool for appraising SRs, was applied to evaluate the methodological quality of the included SRs/MAs. An SR’s validity can be influenced by seven critical items (2, 4, 7, 9, 11, 13, and 15). Based on the adherence to the standard, each item may be classified as “no,” “partial yes,” or “yes.” The overall confidence in the results of SRs/MAs can be divided into four levels: high, moderate, low, or very low.

#### Assessment of Risk of Bias

The ROBIS tool was applied to evaluate the risk of bias of the included SRs/MAs, which was completed in 3 phases: evaluating the consistency between the target problem and the proposed question, identifying concerns in the SR process, and assessing the overall risk of bias (Whiting et al., 2016). The results were judged as “low,” “unclear,” or “high.”

#### Assessment of Reporting Quality

The PRISMA statement, a 27-item checklist, was applied to evaluate the reporting quality of the included SRs/MAs (Moher et al., 2009). Each item was considered “yes” (complete reporting), “partially reported” (partial reporting), or “no” (no reporting) based on the integrity of reporting of the item information.

#### Assessment of Quality of Evidence

The GRADE system was applied to evaluate the evidence quality of the included SRs/MAs, which were downgraded for five aspects: study limitations, inconsistency, indirectness, imprecision, and reporting bias (Atkins et al., 2004). We classified the grade of evidence of an SR into the following four levels: high, moderate, low, and very low.

### Data Synthesis

We used narrative synthesis in this study. The characteristics and results of the included SRs/MAs are presented in Table 1. The dichotomous data were summarized as risk ratio (RR) or odds ratio (OR) with 95% confidence intervals (CI), whereas the continuous data were described as standard mean difference (SMD) or weighted mean difference (WMD) with 95% CI. Additionally, the results of AMSTAR 2, ROBIS, PRISMA, and GRADE are shown in the tables and figures.

**TABLE 1:** Characteristics of the included SRs/MAs.

Study ID	Country	Design	No. of included studies (sample size)	Intervention (dosage, frequency)	Control (dosage, frequency)	Duration of intervention (weeks)	Outcome measures	Data analysis methods	Methodological quality assessment tool	Adverse events
Fang, 2016	China	RCT	16(2,177)	CHM decoction (150–200 ml, 2–3 times/day)	Acitretin (10–40 mg/day); di yin tablet (10–15 tablets/day)	4–12	Total effective rate; PASI score	MA	Modified Jadad	Yes
Zhou, 2020	China	RCT	14(1,334)	CHM fumigation (34–42°C, 20–30 min, 2–3 times/week) + NB-UVB	NB-UVB (311 nm, initial dosage: 0.1–0.5 J/cm^2^; increment regimen: 0.01–0.1 J/cm^2^; maximum dosage: 2–5 J/cm^2^; frequency: 2–3 times/week)	4–8	Total effective rate; cure rate; PASI score	MA	Cochrane	Yes
Li, 2012	China	RCT	8(946)	CHM decoction (150–200 ml, 2–3 times/day)/bath (37–40°C, 20–30 min, 3 times/week) + NB-UVB	NB-UVB (311 nm, initial dosage: 0.2–1 J/cm^2^; increment regimen: 0.1–0.2 J/cm^2^; maximum dosage: 2–2.5 J/cm^2^; frequency: 2–3 times/week)	3–8	Total effective rate; recurrence rate	MA	Cochrane	Yes
Wang, 2019	China	RCT	13(1,116)	CHM decoction (200 ml, 2 times/day)/bath (38–40°C, 30 min, 3 times/week)/xiaoyin granule (3.5 g, 3 times/day)/biejiajian pill (3 g, 2–3 times/day) + conventional Western medicine	Conventional Western medicine: acitretin (20–50 mg/day); calcipotriol ointment (1 time/day); triamcinolone acetonide (1 time/day); vitamin A cream (1 time/day); NB-UVB: (311 nm, initial dosage: 0.1–0.5 J/cm^2^; increment regimen: 0.1–0.15 J/cm^2^; maximum dosage: 2.4 J/cm^2^; frequency: 2–3 times/week)	3–12	Total effective rate (PASI 60)	MA	Cochrane; Jadad; CONSORT	Yes
Wu, 2019	China	RCT	7(660)	Yinxie capsule (1.35–1.8 g, 3 times/day) + acitretin	Acitretin (20 mg/day)	8	Total effective rate (PASI 30); PASI score	MA	Cochrane	Yes
Yang, 2020	China	RCT	20(1,592)	CHM decoction (150 ml, 2 times/day); CHM decoction (100–200 ml, 2–3 times/day) + acitretin	Acitretin (10–80 mg/day)	4–16	Total effective rate; cure rate; PASI score	MA	Cochrane	Yes
Zhang, 2018	China	RCT	17(1920)	CHM decoction (100–200 ml, 2–3 times/day); CHM decoction (100–200 ml, 2–3 times/day) + acitretin	Acitretin (10–50 mg/day)	2–12	Total effective rate; PASI score	MA	Cochrane	Yes
Huang, 2018	China	RCT	8(748)	Total glucosides of paeony capsule (0.6 g 3 times/day) + NB-UVB	NB-UVB (311 nm, initial dosage: 0.1–0.5 J/cm^2^; increment regimen: 0.1 J/cm^2^; maximum dosage: 2.5–3 J/cm^2^; frequency: 2–3 times/week)	6–24	Total effective rate; PASI score	MA	Cochrane	Yes
Guan, 2017	China	RCT	25(3,570)	CHM bath (35–42°C, 15–30 min, 3–7 times/week) + phototherapy	Phototherapy: NB-UVB (311 nm, initial dosage: 0.1–0.5 J/cm^2^; increment regimen: 0.1 J/cm^2^; maximum dosage: 1.5–2.5 J/cm^2^; frequency: 2–7 times/week);	4–8	Total effective rate (PASI 60); recurrence rate; PASI score	MA	Cochrane	Yes
Hao, 2020	China	RCT	20(2,303)	Xiaoyin granule (3.5 g 3 times/day) + acitretin	Acitretin (20–50 mg/day)	4–16	Total effective rate (PASI 30); cure rate (PASI 80); relapse rate; PASI score	MA	Cochrane; modified Jadad	Yes
Feng, 2008	China	RCT	7(626)	CHM decoction (100–150 ml, 2–3 times/day)/xiaoyin granule (3.5 g, 3 times/day) + acitretin	Acitretin (10–30 mg/day)	8–16	Total effective rate	MA	Cochrane	No
Wu, 2015	China	RCT	21(2086)	CHM decoction (100–150 ml, 2 times/day)/bath (20 min, 1–2 times/day)/xiaoyin granule (3.5 g, 3 times/day)/ruizao zhiyang capsule (2–3 capsules, 3 times/day) + acitretin	Acitretin (10–50 mg/day)	8–12	Total effective rate; markedly effective rate; PASI score	MA	Cochrane	No
Zhang, 2016	Australia	RCT	25(2,890)	CHM decoction (150–200 ml, 2 times/day)/kushenin tablet (2 tablets, 3 times/day); CHM decoction (150–200 ml, 2 times/day)/ruizao zhiyang capsule (4 capsules, 3 times/day)/kushenin tablet (2 tablets, 3 times/day)/dahuang zhechong pill (3 g, 3 times/day)/yinxieling tablet (6 tablets, 3 times/day) + Western drugs	Western drugs: acitretin (10–75 mg/day); tazarotene (1 time/day); calcipotriol ointment (2 times/day)	6–12	PASI 60; PASI 90	MA	Cochrane	Yes
Yang, 2015	China	RCT	18(1,416)	CHM decoction (200 ml, 2 times/day)/xiaoyin granule (0.5–1 pack, 2–3 times/day)/wushe jiedu pill (6 g, 3 times/day)/keyin pill (10 g, 2 times/day) + NB-UVB	NB-UVB (311 nm, initial dosage: 0.2–0.7 J/cm^2^; increment regimen: 0.1 J/cm^2^; maximum dosage: 1.6–3 J/cm^2^; frequency: 2–3 times/week)	4–12	PASI 60; PASI 90	MA	Cochrane	Yes

RCT, randomized controlled trial; CHM, Chinese herbal medicine; PASI, psoriasis area and severity index; PASI 30, 60, 80, 90, a PASI score reduction of at least 30, 60, 80, 90%; MA, meta-analysis; NB-UVB, narrowband ultraviolet.

## Results

### Literature Selection

**FIGURE 1:**
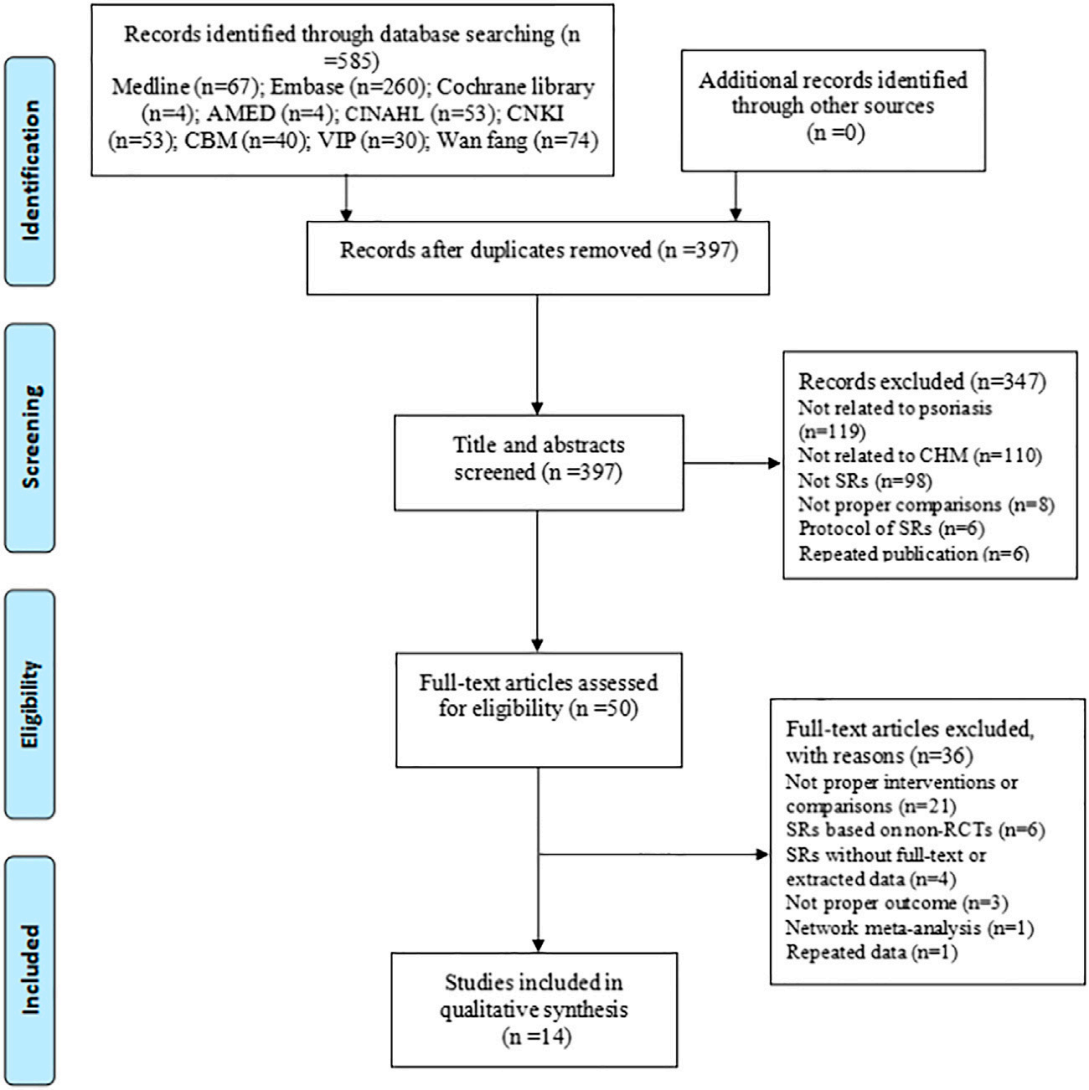
Flow diagram of the study selection process.

A total of 585 articles were identified through our search strategies, and 397 articles were screened for titles and abstracts after removing the duplicates. From these, 50 full-text articles were obtained to assess their eligibility according to the inclusion and exclusion criteria, and subsequently, 14 articles (Feng and Xu, 2008; Li and Liu, 2012; Wu et al., 2015; Yang et al., 2015; Fang et al., 2016; Zhang et al., 2016; Guan et al., 2017; Huang, 2018; Zhang et al., 2018; Wang, 2019; Wu et al., 2019; Hao et al., 2020; Yang et al., 2020; Zhou et al., 2020) were finally included in this overview. The process of study selection is shown in Figure 1.

### Study Characteristics

The 14 included SRs/MAs were published between 2008 and 2020, with 11 articles published in Chinese and three articles (Yang et al., 2015; Zhang et al., 2016; Guan et al., 2017) published in English. The number of RCTs included in the 14 articles ranged from 7 to 25 with a sample size between 660 and 3,570. One article (Fang et al., 2016) compared the efficacy of CHM with that of WM, 10 articles compared the efficacy of CHM plus WM to that of WM, and the remaining three articles (Zhang et al., 2016; Zhang et al., 2018; Yang et al., 2020) conducted both the abovementioned comparisons, in which CHM consisted of oral Chinese decoctions, Chinese patent medicines, Chinese herbal baths, and Chinese medicine fumigations, whereas WM included phototherapy and conventional drugs. Eleven articles used the Cochrane risk of bias tool to evaluate the methodological quality of SRs/MAs, one article (Fang et al., 2016) used a modified Jadad scale, and two articles (Wang, 2019; Hao et al., 2020) used both the aforementioned tools. Total effective rate, cure rate, and markedly effective rate were assessed based on the PASI decline rate. Although the total effective rate was reported in 12 articles, it was only defined in four articles (Guan et al., 2017; Wang, 2019; Wu et al., 2019; Hao et al., 2020), two of them (Guan et al., 2017; Wang, 2019) defined it as the proportion of patients achieving 60% improvement in PASI score (PASI 60), and two articles (Wu et al., 2019; Hao et al., 2020) defined it as PASI 30. The cure rate was reported in three of the articles and was defined as PASI 80 in only one article. Additionally, two articles (Yang et al., 2015; Zhang et al., 2016) assessed the effectiveness of CHM based on PASI 60 and PASI 90, which are usually considered to represent “markedly effective” and “clinical cure” in the Chinese guideline (Zhang et al., 2016). Twelve articles (Li and Liu, 2012; Yang et al., 2015; Fang et al., 2016; Zhang et al., 2016; Guan et al., 2017; Huang, 2018; Zhang et al., 2018; Wang, 2019; Wu et al., 2019; Hao et al., 2020; Yang et al., 2020; Zhou et al., 2020) reported the adverse events, and six articles (Li and Liu, 2012; Yang et al., 2015; Guan et al., 2017; Huang, 2018; Hao et al., 2020; Zhou et al., 2020) conducted a meta-analysis on the incidence of adverse events. The characteristics of the included articles are shown in Table 1.

### Description of the CHM

A total of 110 formulas were found in the intervention group of CHM decoction, and the top eight high-frequency used herbs were used more than 50 times including Radix rehmanniae preparate, Cortex moutan, Radix peoniae rubra, Radix salvia miltiorrhizae, Rhizoma smilacis glabrae, Flos lonicerae, Radix arnebiae seu lithospermi, and Radix glycyrrhizae uralensis. Thirty formulas were applied in the intervention group treated with CHM bath, and the top seven most frequently used herbs were Fructus kochiae scopariae, Cortex dictamni, Radix sophorae flavescentis, Radix salvia miltiorrhizae, Radix angelicae sinensis, Rhizoma smilacis glabrae, and Cortex phellodendri. In the intervention group receiving the CHM fumigation, 13 formulas were used and the herbs that were used more than 5 times consisted of Radix sophorae flavescentis, Cortex dictamni, Flos lonicerae, Radix rehmanniae preparate, Flos chrysanthemi indici, and Radix angelicae sinensis. The details of high-frequently used herbs for psoriasis in the intervention group of CHM decoction, bath, and fumigation are generalized in Table 2. A total of 10 CHM patent medicines were used in these included studies, and the xiaoyin granule was the most commonly used drug. The composition of Chinese patent medicines used in the included studies is presented in Supplementary Material 2.

**TABLE 2:** Details of highly frequently used herbs for psoriasis in the CHM decoction, bath, and fumigation.

Chinese name	Pharmaceutical name	Species	Family
CHM decoction			
Shengdi	Radix rehmanniae preparata	*Rehmannia glutinosa* (Gaertn.) DC.	Orobanchaceae
Mudanpi	Cortex moutan	*Paeonia suffruticosa* Andr.	Paeoniaceae
Chishao	Radix peoniae rubra	*P. veitchii* Lynch	Paeoniaceae
Danshen	Radix salvia miltiorrhizae	*Salvia miltiorrhiza* Bunge	Lamiaceae
Tufuling	Rhizoma smilacis glabrae	*Smilax glabra* Roxb.	Smilacaceae
Jinyinhua	Flos lonicerae	*Lonicera japonica* Thunb.	Caprifoliaceae
Gancao	Radix glycyrrhizae uralensis	*Glycyrrhiza uralensis* Fisch. Ex DC.	Fabaceae
CHM bath			
Difuzi	Fructus kochiae scopariae	*Bassia scoparia* (L.) A.J.Scott	Amaranthaceae
Baixianpi	Cortex dictamni	*Dictamnus dasycarpus* Turcz.	Menispermaceae
Kushen	Radix sophorae flavescentis	*Sophora flavescens* Ait.	Fabaceae
Danshen	Radix salvia miltiorrhizae	*Salvia miltiorrhiza* Bunge	Lamiaceae
Danggui	Radix angelicae sinensis	*Angelica sinensis* (Oliv.) Diels	Apiaceae
Tufuling	Rhizoma smilacis glabrae	*Smilax glabra* Roxb.	Smilacaceae
Huangbo	Cortex phellodendri	*Phellodendron amurense* Rupr.	Rutaceae
CHM fumigation			
Kushen	Radix sophorae flavescentis	*Sophora flavescens* Ait.	Fabaceae
Baixianpi	Cortex dictamni	*Dictamnus dasycarpus* Turcz.	Menispermaceae
Jinyinhua	Flos lonicerae	*Lonicera japonica* Thunb.	Caprifoliaceae
Shengdi	Radix rehmanniae preparata	*Rehmannia glutinosa* (Gaertn.) DC.	Orobanchaceae
Yejuhua	Flos chrysanthemi indici	*Chrysanthemum indicum* L.	Asteraceae
Danggui	Radix angelicae sinensis	*Angelica sinensis* (Oliv.) Diels	Apiaceae

### Methodological Quality of the Included SRs/MAs

The methodological quality of 12 SRs/MAs evaluated by the AMSTAR 2 tool was rated very low, and 2 SRs/MAs were rated low. Significant methodological flaws were found in key items 2 and 7, as well as nonkey items 10, 12, and 16. The details of the AMSTAR 2 assessment of the included SRs/MAs are presented in Table 3.

**TABLE 3:** Results of AMSTAR 2 assessment.

Study ID	Item 1	Item 2	Item 3	Item 4	Item 5	Item 6	Item 7	Item 8	Item 9	Item 10	Item 11	Item 12	Item 13	Item 14	Item 15	Item 16	Overall quality
Fang, 2016	Y	N	Y	Y	N	N	N	N	PY	N	N	N	Y	N	Y	N	Very low
Zhou, 2020	Y	N	Y	PY	Y	Y	N	PY	Y	N	Y	N	N	N	Y	N	Very low
Li, 2012	Y	N	Y	PY	N	N	N	N	PY	N	N	N	N	N	N	N	Very low
Wang, 2019	Y	N	Y	Y	Y	Y	N	Y	Y	N	Y	N	Y	Y	Y	N	Very low
Wu, 2019	Y	N	Y	PY	Y	Y	N	Y	Y	N	N	Y	N	N	Y	N	Very low
Yang, 2020	Y	N	Y	PY	Y	Y	N	PY	Y	N	N	N	N	Y	Y	N	Very low
Zhang, 2018	Y	N	Y	N	N	Y	N	Y	Y	N	N	N	Y	N	N	N	Very low
Huang, 2018	Y	N	Y	PY	Y	Y	N	Y	Y	N	Y	Y	Y	Y	Y	N	Very low
Guan, 2017	Y	N	Y	Y	Y	Y	Y	Y	Y	N	Y	N	Y	Y	Y	Y	Low
Hao, 2020	Y	N	Y	Y	Y	Y	N	Y	Y	N	N	Y	Y	N	Y	N	Very low
Feng, 2008	Y	N	Y	PY	N	N	N	N	Y	N	Y	N	Y	Y	Y	N	Very low
Wu, 2015	Y	N	Y	PY	N	Y	N	Y	Y	N	N	N	Y	Y	Y	N	Very low
Zhang, 2016	Y	N	Y	Y	Y	Y	Y	Y	Y	N	Y	N	Y	Y	Y	Y	Low
Yang, 2015	Y	N	Y	Y	Y	Y	N	Y	Y	N	Y	Y	Y	Y	Y	Y	Very low

Y, yes; PY, partially yes; N, no.

### Risk of Bias of the Included SRs/MAs

The risk of bias of all SRs/MAs in phase 1 (assessing relevance) and domain 1 (study eligibility criteria) evaluated by the ROBIS tool was rated low. Domain 2 assessed the identification and selection of studies, in which 4 articles were rated low risk. Ten articles were rated as low risk of bias in domain 3 (data collection and study appraisal), six articles were rated as low risk of bias in domain 4 (synthesis and findings), and seven articles were rated as low risk of bias in phase 3 (risk of bias in the review). The details of the ROBIS assessment of the included SRs/MAs are shown in Table 4.

**TABLE 4:** Results of ROBIS assessment.

Review	Phase 1	Phase 2				Phase 3
	Assessing relevance	Domain 1: study eligibility criteria	Domain 2: identification and selection of studies	Domain 3: data collection and study appraisal	Domain 4: synthesis and findings	Risk of bias in the review
Fang, 2016	Low	Low	Low	High	High	Low
Zhou, 2020	Low	Low	High	High	High	High
Li, 2012	Low	Low	High	High	High	High
Wang, 2019	Low	Low	High	Low	Low	Low
Wu, 2019	Low	Low	High	Low	High	High
Yang, 2020	Low	Low	High	Low	High	High
Zhang, 2018	Low	Low	High	Low	High	High
Huang, 2018	Low	Low	High	Low	Low	Low
Guan, 2017	Low	Low	Low	Low	Low	Low
Hao, 2020	Low	Low	Low	Low	Low	Low
Feng, 2008	Low	Low	High	High	High	High
Wu, 2015	Low	Low	High	Low	High	Low
Zhang, 2016	Low	Low	Low	Low	Low	Low
Yang, 2015	Low	Low	High	Low	Low	High

### Reporting Quality of the Included SRs/MAs

Table 5 presents the details of the PRISMA checklist of each SR/MA. Twenty-two of 27 items were reported over 70% in the response rate of “Yes,” indicating that the reporting was relatively complete. However, there were also several reporting flaws in the other items. Items 8 (search), 9 (study selection), 23 (additional analyses), and 27 (funding) were inadequately reported (less than 50% in the response rate of “Yes”).

**TABLE 5:** Results of the PRISMA checklist.

Section/topic	Items	Fang, 2016	Zhou, 2020	Li, 2012	Wang, 2019	Wu, 2019	Yang, 2020	Zhang, 2018	Huang, 2018	Guan, 2017	Hao, 2020	Feng, 2008	Wu, 2015	Zhang, 2016	Yang, 2015	Number of yes and partially yes (%)
Title	1. Title	Y	Y	Y	Y	Y	Y	Y	Y	Y	Y	Y	Y	Y	Y	14 (100%)
Abstract	2. Structured summary	PY	Y	PY	Y	Y	Y	Y	Y	Y	Y	Y	Y	PY	Y	14 (100%)
Introduction	3. Rationale	N	Y	N	Y	Y	Y	Y	Y	Y	Y	Y	Y	Y	Y	12 (85.71%)
	4. Objectives	PY	Y	Y	PY	PY	PY	PY	Y	Y	PY	Y	Y	Y	Y	14 (100%)
Methods	5. Protocol and registration	N	N	N	N	N	N	N	N	N	N	N	N	N	N	0 (0%)
	6. Eligibility criteria	Y	Y	PY	Y	Y	Y	Y	Y	Y	Y	Y	Y	Y	Y	14 (100%)
	7. Information sources	Y	Y	Y	Y	Y	Y	Y	Y	Y	Y	Y	Y	Y	Y	14 (100%)
	8. Search	N	N	N	N	N	N	Y	N	N	Y	N	N	Y	Y	4 (28.57%)
	9. Study selection	N	Y	N	Y	PY	Y	N	PY	PY	Y	N	N	Y	N	8 (57.14%)
	10. Data collection process	Y	Y	N	Y	Y	Y	Y	Y	Y	Y	N	Y	Y	Y	12 (85.71%)
	11. Data items	Y	N	N	Y	Y	Y	Y	Y	Y	Y	N	Y	N	Y	10 (71.43%)
	12. Risk of bias in individual studies	PY	Y	Y	Y	Y	Y	Y	Y	Y	Y	Y	Y	Y	Y	14 (100%)
	13. Summary measures	Y	Y	Y	Y	Y	Y	Y	Y	Y	Y	Y	Y	Y	Y	14 (100%)
	14. Synthesis of results	Y	Y	N	Y	Y	Y	Y	Y	Y	Y	Y	Y	Y	N	12 (85.71%)
	15. Risk of bias across studies	Y	Y	N	Y	Y	Y	N	Y	N	Y	Y	Y	N	Y	10 (71.43%)
	16. Additional analyses	Y	Y	N	Y	Y	Y	Y	N	Y	Y	Y	Y	Y	Y	12 (85.71%)
Results	17. Study selection	Y	Y	PY	Y	Y	Y	Y	Y	Y	Y	PY	Y	Y	Y	14 (100%)
	18. Study characteristics	PY	Y	N	Y	Y	Y	Y	Y	Y	Y	PY	Y	Y	PY	13 (92.86%)
	19. Risk of bias within studies	PY	Y	PY	Y	Y	Y	Y	Y	Y	Y	PY	Y	Y	Y	14 (100%)
	20. Results of individual studies	Y	Y	N	Y	Y	Y	Y	Y	Y	Y	Y	Y	N	Y	12 (85.71%)
	21. Synthesis of results	Y	Y	Y	Y	Y	Y	Y	Y	Y	Y	Y	Y	Y	Y	14 (100%)
	22. Risk of bias across studies	Y	Y	N	Y	Y	Y	N	Y	Y	Y	Y	Y	N	Y	11 (78.57%)
	23. Additional analyses	N	N	N	N	Y	N	Y	Y	Y	Y	N	N	Y	Y	7 (50%)
Discussion	24. Summary of evidence	Y	PY	PY	PY	PY	Y	Y	PY	Y	PY	Y	Y	Y	Y	14 (100%)
	25. Limitations	Y	Y	Y	Y	Y	Y	N	Y	Y	Y	Y	N	Y	Y	12 (85.71%)
	26. Conclusions	Y	Y	Y	Y	Y	PY	Y	Y	Y	Y	Y	Y	Y	Y	14 (100%)
Funding	27. Funding	N	PY	N	N	PY	PY	N	N	Y	PY	N	N	Y	Y	7 (50%)
PRISMA score	—	18.5	22	10.5	22	23	22.5	20.5	22	23.5	24.5	18.5	21	21.5	23.5	

Y, yes; PY, partially yes; N, no.

### Efficacy and Safety of CHM for Psoriasis

The outcomes of each SR/MA are summarized and shown in Table 6.

**TABLE 6:** Summary of evidence.

Outcomes	Study ID	Synthesis of results	Total patient number in the treatment or control group	Number of studies
CHM vs. WM (including phototherapy)				
Total effective rate	Fang, 2016	OR = 3.00, 95% CI (2.33, 3.86), I^2^ = 23%, *p* < 0.00001	1,279/898	16
	Yang, 2020	OR = 3.33, 95% CI (1.98, 5.66), I^2^ = 0%, *p* < 0.00001	288/286	8
	Zhang, 2018	OR = 1.94, 95% CI (1.34, 2.80), I^2^ = 40%, *p* < 0.0001	745/676	13
Cure rate	Yang, 2020	OR = 2.85, 95% CI (1.90, 4.29), I^2^ = 0%, *p* < 0.00001	288/286	8
PASI 60	Zhang, 2016	RR = 0.99, 95% CI (0.95, 1.04), I^2^ = 0%, *p* > 0.05	829/667	13
PASI 90	Zhang, 2016	RR = 1.00, 95% CI (0.86, 1.16), I^2^ = 0%, *p* > 0.05	795/634	12
PASI score	Fang, 2016	MD = −1.43, 95% CI (−2.56, −0.29), I^2^ = 91%, *p* = 0.01	565/466	7
	Yang, 2020	MD = −2.16, 95% CI (−3.19, −1.12), I^2^ = 92%, *p* < 0.00001	239/239	7
	Zhang, 2018	MD = −2.29, 95% CI (−4.02, −0.57), I^2^ = 0%, *p* < 0.00001	356/361	6
CHM + WM vs. WM				
Total effective rate	Zhou, 2020	OR = 4.17, 95% CI (3.05, 5.70), I^2^ = 0%, *p* < 0.0001	668/666	14
	Li, 2012	RR = 1.26, 95% CI (1.18, 1.35), I^2^ = 1%, *p* < 0.001	473/473	8
	Wang, 2019	RR = 1.26, 95% CI (1.19, 1.33), I^2^ = 0%, *p* < 0.00001	573/540	13
	Wu, 2019	RR = 1.15, 95% CI (1.04, 1.28), I^2^ = 74%, *p* = 0.007	328/332	7
	Yang, 2020	OR = 4.03, 95% CI (2.48, 6.56), I^2^ = 0%, *p* < 0.00001	342/335	9
	Zhang, 2018	OR = 2.67, 95% CI (1.55, 4.60), I^2^ = 0%, *p* < 0.0001	228/216	3
	Huang, 2018	OR = 3.07, 95% CI (1.95, 4.82), I^2^ = 0%, *p* < 0.00001	378/370	8
	Guan, 2017	OR = 3.25, 95% CI (2.69, 3.93), I^2^ = 0%, *p* < 0.00001	1839/1731	25
	Hao, 2020	OR = 3.55, 95% CI (2.76, 4.57), I^2^ = 0%, *p* < 0.00001	1,143/1,080	19
	Feng, 2008	OR = 3.13, 95% CI (1.77, 5.55), I^2^ = 0%, *p* < 0.0001	356/270	7
	Wu, 2015	RR = 1.10, 95% CI (1.04, 1.16), I^2^ = 78%, *p* < 0.01	1,209/899	21
Cure rate	Zhou, 2020	OR = 3.26, 95% CI (2.29, 4.63), I^2^ = 46%, *p* < 0.00001	668/666	14
	Yang, 2020	OR = 2.15, 95% CI (1.55, 2.98), I^2^ = 0%, *p* < 0.00001	342/335	9
	Hao, 2020	OR = 2.47, 95% CI (2.05, 2.98), I^2^ = 29%, *p* < 0.00001	1,091/1,028	18
Markedly effective rate	Wu, 2015	RR = 1.34, 95% CI (1.20, 1.51), I^2^ = 75%, *p* < 0.01	1,027/829	19
PASI 60	Zhang, 2016	RR = 1.40, 95% CI (1.31, 1.50), I^2^ = 0%, *p* < 0.05	824/807	17
	Yang, 2015	RR = 1.35, 95% CI (1.26, 1.45), I^2^ = 4%, *p* < 0.00001	695/647	17
PASI 90	Zhang, 2016	RR = 1.55, 95% CI (1.37, 1.75), I^2^ = 0%, *p* < 0.05	824/807	17
	Yang, 2015	RR = 1.71, 95% CI (1.45, 2.01), I^2^ = 0%, *p* < 0.00001	695/647	17
PASI score	Zhou, 2020	MD = −2.25, 95% CI (−3.69, −0.82), I^2^ = 88%, *p* = 0.002	155/157	4
	Wu, 2019	MD = −2.34, 95% CI (−2.77, −1.91), I^2^ = 0%, *p* < 0.00001	244/244	5
	Yang, 2020	MD = −3.27, 95% CI (−4.90, −1.65), I^2^ = 98%, *p* < 0.00001	255/252	5
	Zhang, 2018	MD = −2.78, 95% CI (−3.86, −1.70), I^2^ = 86%, *p* < 0.00001	96/96	2
	Huang, 2018	Decline of PASI score: MD = 3.03, 95% CI (2.21, 3.85), I^2^ = 62%, *p* < 0.00001	378/370	8
	Guan, 2017	MD = −3.46, 95% CI (−4.66, −2.26), I^2^ = 93%, *p* < 0.00001	869/793	12
	Hao, 2020	MD = −2.21, 95% CI (−2.98, −1.43), I^2^ = 68%, *p* < 0.00001	192/192	4
	Wu, 2015	MD = −2.35, 95% CI (−3.64, −1.06), I^2^ = 83%, *p* = 0.0003	129/109	3
Recurrence rate	Li, 2012	RR = 0.26, 95% CI (0.11, 0.60), I^2^ = 1%, *p* = 0.002	124/131	2
	Guan, 2017	OR = 0.27, 95% CI (0.18, 0.42), I^2^ = 34%, *p* < 0.00001	289/186	5
	Hao, 2020	OR = 0.41, 95% CI (0.24, 0.69), I^2^ = 0%, *p* = 0.0008	228/228	5
Adverse events incidence	Zhou, 2020	OR = 0.51, 95% CI (0.27, 0.95), I^2^ = 67%, *p* = 0.04	605/603	12
	Li, 2012	RR = 0.88, 95% CI (0.42, 1.84), I^2^ = 70%, *p* = 0.74	395/395	7
	Huang, 2018	OR = 1.30, 95% CI (0.80, 2.12), I^2^ = 0%, *p* = 0.29	378/370	8
	Guan, 2017	OR = 0.59, 95% CI (0.33, 1.05), I^2^ = 75%, *p* = 0.07	1,010/939	13
	Hao, 2020	OR = 0.68, 95% CI (0.38, 1.20), I^2^ = 64%, *p* = 0.18	440/426	8
	Yang, 2015	RR = 0.66, 95% CI (0.46, 0.96), I^2^ = 53%, *p* < 0.05	464/428	12

CHM, Chinese herbal medicine; WM, Western medicine; RR, risk ratio; OR, odds ratio; MD, mean difference; CI, confidence interval.

#### Efficacy Evaluation

##### CHM vs. WM

Four SRs/MAs (Fang et al., 2016; Zhang et al., 2016; Zhang et al., 2018; Yang et al., 2020) compared the effects of CHM and WM, of which 3 articles (Fang et al., 2016; Zhang et al., 2018; Yang et al., 2020) reported the total effective rate, indicating that CHM had a higher total effective rate than WM (*p* < 0.05). Only one article (Yang et al., 2020) reported the cure rate, and the result showed that CHM significantly increases the cure rate when compared with WM [OR = 2.85, 95% CI (1.90, 4.29), I^2^ = 0%, *p* < 0.00001]. The PASI score was assessed in three articles (Fang et al., 2016; Zhang et al., 2018; Yang et al., 2020), and all of them demonstrated that CHM significantly reduced the PASI score compared with WM (*p* < 0.05). However, one article (Zhang et al., 2016) showed no significant difference between CHM and WM for achieving PASI 60 or PASI 90 [RR = 0.99, 95% CI (0.95, 1.04), I^2^ = 0%, *p* > 0.05; RR = 1.00, 95% CI (0.86, 1.16), I^2^ = 0%, *p* > 0.05].

##### CHM Plus WM vs. WM

Thirteen SRs/MAs (Feng and Xu, 2008; Li and Liu, 2012; Wu et al., 2015; Yang et al., 2015; Zhang et al., 2016; Guan et al., 2017; Huang, 2018; Zhang et al., 2018; Wang, 2019; Wu et al., 2019; Hao et al., 2020; Yang et al., 2020; Zhou et al., 2020) compared the effects of CHM plus WM with WM alone. Eleven articles (Feng and Xu, 2008; Li and Liu, 2012; Wu et al., 2015; Guan et al., 2017; Huang, 2018; Zhang et al., 2018; Wang, 2019; Wu et al., 2019; Hao et al., 2020; Yang et al., 2020; Zhou et al., 2020) found a greater total effective rate in the combined therapy group than in the WM group (*p* < 0.05). Three articles (Hao et al., 2020; Yang et al., 2020; Zhou et al., 2020) assessed the cure rate, and they showed that combined therapy was superior to WM alone (*p* < 0.05). Only one article (Wu et al., 2015) analyzed the markedly effective rate, which was higher in the combined therapy group than in the WM group [RR = 1.34, 95% CI (1.20, 1.51), I^2^ = 75%, *p* < 0.01]. Two articles (Yang et al., 2015; Zhang et al., 2016) indicated that combined therapy had a superior effect on achieving PASI 60 and PASI 90 than WM alone. The PASI score was used to assess the effects of CHM in eight articles (Wu et al., 2015; Guan et al., 2017; Huang, 2018; Zhang et al., 2018; Wu et al., 2019; Hao et al., 2020; Yang et al., 2020; Zhou et al., 2020), and all of them revealed that the combined therapy significantly decreased the PASI score compared with WM alone (*p* < 0.05). Three articles (Li and Liu, 2012; Guan et al., 2017; Hao et al., 2020) showed a lower recurrence rate in the combined therapy than WM (*p* < 0.05).

#### Adverse Events

Twelve SRs/MAs reported adverse events, and four of them (Fang et al., 2016; Zhang et al., 2016; Zhang et al., 2018; Yang et al., 2020) stated that the common adverse events in the CHM group were diarrhea, vomiting, nausea, and abdominal discomfort, which were slightly less severe than those in the WM group. Thirteen SRs/MAs (Feng and Xu, 2008; Li and Liu, 2012; Wu et al., 2015; Yang et al., 2015; Zhang et al., 2016; Guan et al., 2017; Huang, 2018; Zhang et al., 2018; Wang, 2019; Wu et al., 2019; Hao et al., 2020; Yang et al., 2020; Zhou et al., 2020) reported adverse events related to the combined therapy, including xerosis cutis, dry mouth, skin erythema, pruritus, gastrointestinal discomfort, elevated transaminase, and hyperlipidemia. One study (Zhou et al., 2020) pointed out that skin erythema and pruritus might be related to ultraviolet radiation, whereas another study (Yang et al., 2020) mentioned that elevated transaminase may be associated with acitretin in the combined therapy group. Six studies (Li and Liu, 2012; Yang et al., 2015; Guan et al., 2017; Huang, 2018; Hao et al., 2020; Zhou et al., 2020) conducted an MA on the total incidence of adverse events, of which four studies (Li and Liu, 2012; Guan et al., 2017; Huang, 2018; Hao et al., 2020) showed no significant difference between the combined therapy and WM (*p* > 0.05).

### Evidence Quality of the Included SRs/MAs

The details of the GRADE assessment of 45 outcomes in 14 SRs/MAs are presented in Table 7. The results indicated that 15 (15/45, 33.33%), 16 (16/45, 35.56%), and 14 (14/45, 31.11%) outcomes were rated as moderate, low, and very low quality, respectively. No outcome of high-quality evidence was found. The experimental designs of all the original RCTs had a serious risk of bias, which was the main factor for the downgrading of the evidence quality. The secondary factors for downgrading were publication bias (23/45, 51.11%), inconsistency (17/45, 37.78%), and imprecision (8/45, 17.78%).

**TABLE 7:** Results of evidence quality.

Outcomes	Study ID	Studies (participants)	Risk of bias	Inconsistency	Indirection	Imprecision	Publication bias	Quality of evidence
CHM vs. WM								
Total effective rate	Fang, 2016	16 (2,177)	−1^①^	0	0	0	−1^④^	Low
	Yang, 2020	8 (574)	−1^①^	0	0	0	−1^④^	Low
	Zhang, 2018	13(1,421)	−1^①^	0	0	0	0	Moderate
Cure rate	Yang, 2020	8(574)	−1^①^	0	0	0	−1^④^	Low
PASI 60	Zhang, 2016	13(1,496)	−1^①^	0	0	0	0	Moderate
PASI 90	Zhang, 2016	12(1,429)	−1^①^	0	0	0	0	Moderate
PASI score	Fang, 2016	7 (1,037)	−1^①^	−1^②^	0	0	0	Low
	Yang, 2020	7(478)	−1^①^	−1^②^	0	0	−1^④^	Very low
	Zhang, 2018	6(717)	−1^①^	0	0	0	0	Moderate
CHM + WM vs. WM								
Total effective rate	Zhou, 2020	14(1,334)	−1^①^	0	0	0	−1^④^	Low
	Li, 2012	8(946)	−1^①^	0	0	0	0	Moderate
	Wang, 2019	13(1,113)	−1^①^	0	0	0	0	Moderate
	Wu, 2019	7(660)	−1^①^	−1^②^	0	0	−1^④^	Very low
	Yang, 2020	9(677)	−1^①^	0	0	0	−1^④^	Low
	Zhang, 2018	3(444)	−1^①^	0	0	0	−1^⑤^	Low
	Huang, 2018	8(748)	−1^①^	0	0	0	−1^④^	Low
	Guan, 2017	25(3,570)	−1^①^	0	0	0	−1^④^	Low
	Hao, 2020	19(2,223)	−1^①^	0	0	0	0	Moderate
	Feng, 2008	7(626)	−1^①^	0	0	0	0	Moderate
	Wu, 2015	21(2,108)	−1^①^	−1^②^	0	0	−1^④^	Very low
Cure rate	Zhou, 2020	14(1,334)	−1^①^	0	0	0	0	Moderate
	Yang, 2020	9(677)	−1^①^	0	0	0	−1^④^	Low
	Hao, 2020	18(2,119)	−1^①^	0	0	0	0	Moderate
Markedly effective rate	Wu, 2015	19(1,360)	−1^①^	−1^②^	0	0	−1^④^	Very low
PASI 60	Zhang, 2016	17(1,631)	−1^①^	0	0	0	0	Moderate
	Yang, 2015	17(1,342)	−1^①^	0	0	0	−1^④^	Low
PASI 90	Zhang, 2016	17(1,631)	−1^①^	0	0	0	0	Moderate
	Yang, 2015	17(1,342)	−1^①^	0	0	0	−1^④^	Low
PASI score	Zhou, 2020	4(312)	−1^①^	−1^②^	0	−1^③^	−1^⑤^	Very low
	Wu, 2019	5(488)	−1^①^	0	0	0	0	Moderate
	Yang, 2020	5(507)	−1^①^	−1^②^	0	0	−1^④^	Very low
	Zhang, 2018	2(192)	−1^①^	−1^②^	0	−1^③^	−1^⑤^	Very low
	Huang, 2018	8(748)	−1^①^	−1^②^	0	0	−1^④^	Very low
	Guan, 2017	12(1,662)	−1^①^	−1^②^	0	0	0	Low
	Hao, 2020	4(384)	−1^①^	−1^②^	0	−1^③^	−1^⑤^	Very low
	Wu, 2015	3(238)	−1^①^	−1^②^	0	−1^③^	−1^⑤^	Very low
Recurrence rate	Li, 2012	2(255)	−1^①^	0	0	−1^③^	−1^⑤^	Very low
	Guan, 2017	5(475)	−1^①^	0	0	0	0	Moderate
	Hao, 2020	5(456)	−1^①^	0	0	0	0	Moderate
Adverse events incidence	Zhou, 2020	12(1,208)	−1^①^	−1^②^	0	0	0	Low
	Li, 2012	7(790)	−1^①^	−1^②^	0	−1^③^	0	Very low
	Huang, 2018	8(748)	−1^①^	0	0	−1^③^	−1^④^	Very low
	Guan, 2017	13(1949)	−1^①^	−1^②^	0	−1^③^	0	Very low
	Hao, 2020	8(1,014)	−1^①^	−1^②^	0	0	0	Low
	Yang, 2015	12(892)	−1^①^	−1^②^	0	0	0	Low

CHM, Chinese herbal medicine; WM, Western medicine; PASI, psoriasis area and severity index; PASI 60, 90, a PASI score reduction of at least 60%, 90%. ^①^The included studies have a large bias in methodology such as randomization, allocation concealment, and blinding. ^②^The confidence interval overlaps less or the I2 value of the combined results was larger. ^③^The sample size from the included studies does not meet the optimal sample size or the 95% confidence interval crosses the invalid line. ^④^The funnel chart is asymmetry. ^⑤^Fewer studies were included, and their results were all positive, which may result in a large publication bias.

## Discussion

### Summary of the Main Findings

This overview included 14 SRs/MAs published between 2008 and 2020, with 8 (8/14, 57.14%) reviews (Guan et al., 2017; Huang, 2018; Zhang et al., 2018; Wang, 2019; Wu et al., 2019; Hao et al., 2020; Yang et al., 2020; Zhou et al., 2020) published in the last 5 years, indicating that there is increasing attention on the effectiveness and safety of CHM for the treatment of psoriasis. Two main findings were found in our overview.

The first one is that there is considerable room to address the risk of bias and improve the methodological and reporting quality of the included SRs/MAs. The methodological quality of all the articles evaluated by the AMSTAR 2 tool was rated very low or low, and the following main flaws were noted: 1) the lack of an SR protocol or the registration of the protocol may have affected their standardization and the thoroughness and increased the possibility of selective reporting bias; 2) missing lists of excluded studies with explanations may have decreased the transparency of the SRs and influenced the confidence of results; and 3) missing statements of the funding source or conflicts of interest may make it difficult for the evidence users to judge the impact of the abovementioned factors on their results. All the abovementioned deficiencies in the methodology limited the validity of the SRs. With the ROBIS tool, the incomplete literature search, improper study selection, inappropriate methods of data synthesis, and inadequate explanation of the risk of bias in the discussion were the main factors resulting in the high risk of bias, which may have affected the reliability of the current evidence. Pertaining to the results of the PRISMA checklist, we found that the detailed search strategies, the complete processes of literature screening, the additional analyses, and the sources of funding were not well reported. Additionally, no SR reported the details of the study protocol or the protocol registration. All the aforementioned flaws of reporting may have influenced the clarity and transparency in how SRs are conducted (Liberati et al., 2009).

The second main finding of our evaluation is that the moderate-quality evidence supported the potential benefits of CHM for patients with psoriasis, but caution is required when recommending the CHM as a complementary intervention for psoriasis. Considering the results of GRADE evaluation in this overview, the moderate-quality evidence revealed that CHM combined with WM can demonstrate the following: 1) a significantly increased total effective rate and cure rate; 2) a significantly achieved higher response of PASI 60 and PASI 90 that are usually considered to represent “markedly effective” and “clinical cure” in the Chinese guidelines; 3) a significantly reduced PASI score and recurrence rate when compared to WM alone, which can provide clinicians with a definite reference point; and 4) more treatment options for patients with psoriasis. These findings indicated that CHM seemed to be a promising intervention in the adjuvant treatment of psoriasis. However, the findings on the effects of CHM used alone were inconsistent among the four articles included (Fang et al., 2016; Zhang et al., 2016; Zhang et al., 2018; Yang et al., 2020). In regards to the evaluation of the safety of CHM for psoriasis, two SRs/MAs showed that the combined therapy significantly reduced the incidence of adverse events when compared with the WM alone, whereas four SRs/MAs (Li and Liu, 2012; Guan et al., 2017; Huang, 2018; Hao et al., 2020) found no difference between the two groups. These findings indicated that CHM was a relatively safe treatment for psoriasis, but the standard of evidence was graded as low or very low.

In addition to the aforementioned unsatisfactory methodological quality and potential risk of bias, the low levels of overall evidence and various efficacy evaluations were also the factors affecting the conclusion’s credibility, and caution is required when interpreting the results. In this overview, no high-quality evidence was found, and most of the outcomes (30/45, 66.67%) were rated low or very low quality. The main factors for the downgrade of evidence quality were the study limitations and the publication biases. Most of the original RCTs of CHM in treating psoriasis did not clearly describe the method of random sequence generation, allocation concealment, and blind method, which may have affected the argumentation strength of the SRs/MAs. The publication biases were mostly caused by the asymmetry of the funnel chart and the small number of included RCTs with positive results. This increased the risk of overestimating the effect value and resulted in a large disparity between the conclusions obtained by the SRs/MAs and the real situation (Guyatt et al., 2011).

Furthermore, several problems of the efficacy evaluation in the included articles that cannot be ignored were as follows: 1) the assessment of clinical effectiveness or cure rate in all the original studies was based on the improvement rate of the PASI score, but these evaluation criteria were missing or inconsistent in many SRs/MAs; 2) the treatment courses of the original studies included in the 13 SRs/MAs (Feng and Xu, 2008; Li and Liu, 2012; Wu et al., 2015; Yang et al., 2015; Fang et al., 2016; Zhang et al., 2016; Guan et al., 2017; Huang, 2018; Zhang et al., 2018; Wang, 2019; Hao et al., 2020; Yang et al., 2020; Zhou et al., 2020) were different; however, only three articles (Yang et al., 2015; Zhang et al., 2016; Zhang et al., 2018) conducted subgroup analysis according to the different treatment courses. These two points highlight the possibilities of heterogeneity between studies, and combining highly heterogeneous results may affect the authenticity of the SRs/MAs; and 3) three articles (Li and Liu, 2012; Guan et al., 2017; Hao et al., 2020) conducted an MA of the recurrence rates, but the original studies included in these MAs had some deficiencies such as different follow-up times and undefined recurrence criteria, which reduced the conviction of the conclusion.

### Implications for Future Study

The overall quality of SRs/MAs is closely related to the methodological and reporting quality of the original studies included. Therefore, rigorously designed multicenter, large-sample RCTs should be carried out in the future. The evaluation of efficacy in the original RCTs revealed that the total effective rate was usually defined as PASI 60, whereas PASI 75 was internationally considered treatment success. To better reflect the efficacy of the interventions and to enhance the international recognition of the evidence of CHM in the treatment of psoriasis, future studies should choose the evaluation indices of efficacy that are recommended by international guidelines or by expert consensuses, such as PASI 75, PASI 90, physician’s global assessment, and body surface area (van der Kraaij et al., 2019; Svoboda et al., 2020). When evaluating the effects of treatment, we should not only consider the abovementioned evaluation indices reported by the physicians to quantify the removal of skin lesions but also take into account the assessment of other conditions (such as quality of life) that affect the efficacy and prognosis of patients with psoriasis. Thus, the use of evaluation indices that can be reported by the patients, such as the Dermatology Life Quality Index, Psoriasis Disability Index, Psoriasis Symptom Inventory, and 36-item short-form health survey, can comprehensively evaluate the efficacy of interventions (Lizán et al., 2019; Svoboda et al., 2020). Additionally, most of the original RCTs either did not mention follow-up or just had a short follow-up. Considering that psoriasis is prone to relapse and that the disease course is long, future studies should extend the follow-up period to further assess the long-term effectiveness of CHM for treating psoriasis to adequately represent the therapeutic advantages of CHM.

Moreover, several suggestions for future directions of SRs are as follows: 1) to achieve a high quality of evidence, future SRs are recommended to be conducted and reported using the AMSTAR 2 tool, ROBIS tool, and PRISMA statement. Although the AMSTAR 2 is a common tool for evaluating the methodological quality of SRs, it cannot assess the risk of bias (Shea et al., 2017). Thus, researchers should also strictly follow the requirement of the ROBIS tool to conduct SRs to minimize the risk of bias; 2) to reduce the potential selection bias, registering the SRs in advance or publishing the protocol, conducting a comprehensive literature search (including gray literature), and listing the excluded studies with explanation are recommended; 3) to boost the reliability of the results, the subgroup or sensitivity analysis should be carried out when the high heterogeneity is found; 4) to promote the use of evidence by the clinicians, the GRADE system should be applied to evaluate the evidence of SR; and 5) to facilitate the evidence users in understanding the impact of the funding sources and conflicts of interest on the results, these factors should be disclosed in the article.

### Strength and Limitations

Our study is the first overview of SRs/MAs to assess the evidence of CHM for treating psoriasis by using the AMSTAR 2, ROBIS, PRISMA, and GRADE. Moreover, we conducted this overview based on the published protocol to reduce the risk of bias as much as possible. However, this overview had some limitations that need to be taken into account. First, the individual effects of systemic use or topical applications of CHM were not distinguished in this overview, which may influence the establishment of the evidence for clinical use. Second, the evaluators may have had differences in subjective evaluation, resulting in bias and influencing the research findings. Finally, the generally low quality of methodology and evidence and the wide disparities (such as treatment course and efficacy criteria) of interventions and outcomes in the included SRs/MAs were found, and the conclusions should be interpreted with caution.

## Conclusion

This overview revealed that CHM seems to be a beneficial and relatively safe intervention in the adjuvant treatment for psoriasis. However, because of the generally low quality of methodology and evidence, we should be cautious about the conclusion. To provide convincing evidence for researchers and clinicians in this field, high-quality RCTs on CHM for treating psoriasis should be carried out in the future, with a simultaneous improvement in the methodological and reporting quality of SRs.

## Data Availability

The original contributions presented in the study are included in the article/Supplementary Material; further inquiries can be directed to the corresponding authors.

## Abbreviations

AMSTAR, A Measurement Tool to Assess Systematic Reviews; CHM, Chinese herbal medicine; CI, confidence intervals; GRADE, Grades of Recommendations, Assessment, Development and Evaluation; OR, odds ratio; PASI, psoriasis area and severity index; PRISMA, Preferred Reporting Items for Systematic Reviews and Meta-analyses; RCTs, randomized controlled trials; ROBIS, Risk of Bias in Systematic Reviews; RR, risk ratio; SMD, standard mean difference; SRs/MAs, systematic reviews/meta-analyses; WM, Western medicine; WMD, weighted mean difference.
