# Supplementary material for: Benefits and Safety of Chinese Herbal Medicine in Treating Psoriasis: An Overview of Systematic Reviews
Source: Front Pharmacol. 2021 Jul 1;12:680172. doi: 10.3389/fphar.2021.680172 (PMC8281221; doi:10.3389/fphar.2021.680172)
Supplement: Supplementary file 1 [file Table1.DOCX]

Supplementary material 1. Search strategies for each database.

Medline via OVID

| Number | Search items | Number of retrieved articles |
| --- | --- | --- |
| #1 | exp Psoriasis/ | 40848 |
| #2 | (psoriasis or psoriases or psoria*).ti,ab. | 50275 |
| #3 | #1 or #2 | 56454 |
| #4 | exp Medicine, Chinese Traditional/ | 19814 |
| #5 | exp Drugs, Chinese Herbal/ | 44676 |
| #6 | (herb* or plant or prescrip* or decoction* or formul*).ti,ab. | 887499 |
| #7 | ((patent or proprietary or herbal) adj medicine).ti,ab. | 11858 |
| #8 | (chinese adj2 (medicine or drug$ or medica)).ti,ab. | 33055 |
| #9 | #4 or #5 or #6 or #7 or #8 | 934676 |
| #10 | meta-analysis.pt. | 125088 |
| #11 | systematic review.ti,ab. | 176234 |
| #12 | meta-analy$.ti,ab. | 191240 |
| #13 | meta analys$.ab,ti. | 188186 |
| #14 | systematic review.pt. | 143282 |
| #15 | #10 or #12 or #13 or #14 | 317478 |
| #16 | #3 and #9 and #15 | 67 |

Embase

| Number | Search items | Number of retrieved articles |
| --- | --- | --- |
| #1 | 'psoriasis'/exp | 101928 |
| #2 | psoriasis OR psoriases OR psoria*:ab,ti | 99544 |
| #3 | #1 or #2 | 104256 |
| #4 | 'chinese medicine'/exp | 54582 |
| #5 | herb* OR plant OR prescrip* OR decoction* OR formul*:ti,ab | 1893772 |
| #6 | 'patent medicine' OR 'proprietary medicine' OR 'herbal medicine':ab,ti | 16464 |
| #7 | chinese NEAR/2 (medicine OR drug$ OR medica) | 188554 |
| #8 | #4 or #5 or #6 or #7 | 2010690 |
| #9 | 'systematic review':ti,ab | 214729 |
| #10 | 'meta analysis':ti,ab | 211295 |
| #11 | 'meta analysis':de | 245049 |
| #12 | 'systematic review':de | 300309 |
| #13 | #9 or #10 or #11 or #12 | 494757 |
| #14 | #3 and #8 and #13 | 260 |

Cochrane Library (Cochrane Reviews).

| Number | Search items | Number of retrieved articles |
| --- | --- | --- |
| #1 | MeSH descriptor: [Psoriasis] explode all trees | 19 |
| #2 | (psoriasis or psoriases or psoria*):ti,ab,kw | 31 |
| #3 | #1 or #2 | 31 |
| #4 | MeSH descriptor: [Medicine, Chinese Traditional] explode all trees | 6 |
| #5 | MeSH descriptor: [Drugs, Chinese Herbal] explode all trees | 95 |
| #6 | (herb* or plant or prescrip* or decoction* or formul*):ti,ab,kw | 1502 |
| #7 | (chinese near/2 (medicine or drug$ or medica)):ti,ab,kw | 99 |
| #8 | ((patent or proprietary or herbal) near/1 medicine):ti,ab,kw | 65 |
| #9 | #4 or #5 or #6 or #7 or #8 | 1524 |
| #10 | #3 and #9 | 4 |

CINAHL via EBSCO.

| Number | Search items | Number of retrieved articles |
| --- | --- | --- |
| S1 | SU psoriasis OR (psoriasis or psoriases or psoria*) | 11195 |
| S2 | (herb* or plant or prescrip* or decoction* or formul*) OR ((patent or proprietary or herbal) N0 medicine) OR (chinese N1 (medicine or drug* or medica or bath)) | 231689 |
| S3 | PT (systematic review or meta-analysis) OR systematic review* OR meta analy* | 190854 |
| S4 | S1 AND S2 AND S3 | 53 |

AMED via EBSCO.

| Number | Search items | Number of retrieved articles |
| --- | --- | --- |
| S1 | SU psoriasis OR (psoriasis or psoriases or psoria*) | 382 |
| S2 | (herb* or plant or prescrip* or decoction* or formul*) OR ((patent or proprietary or herbal) N0 medicine) OR (chinese N1 (medicine or drug* or medica or bath)) | 48579 |
| S3 | systematic review* OR meta analy* | 6267 |
| S4 | S1 AND S2 AND S3 | 4 |

CBM

("系统评价"[全部字段:智能] OR "荟萃分析"[全部字段:智能] OR "Meta"[全部字段:智能]) AND (("中药"[全部字段:智能] OR "草药"[全部字段] OR "中成药"[全部字段:智能] OR "中医药"[全部字段:智能]) OR ("中草药"[不加权:扩展])) AND (("银屑病"[全部字段:智能] OR "白疕"[全部字段:智能] OR "牛皮癣"[全部字段:智能]) OR ("银屑病"[不加权:扩展])) 40

CNKI

SU=('银屑病'+'白疕'+'牛皮癣') AND SU=('中药'+'中医药'+'草药'+'中成药') AND SU=('系统评价'+'荟萃分析'+'Meta') 53

Wang fang database

主题:(银屑病 or 白疕 or 牛皮癣) and 主题:(中药 or 中医药 or 中成药 or 草药) and 主题:(系统评价 or 荟萃分析 or Meta) 74

VIP

任意字段:(银屑病+白疕+牛皮癣) and 任意字段:(中医+中药+中成药+草药) and 任意字段:(系统评价+荟萃分析+Meta) 30
