# Supplementary material for: Benefits and Safety of Chinese Herbal Medicine in Treating Psoriasis: An Overview of Systematic Reviews
Source: Front Pharmacol. 2021 Jul 1;12:680172. doi: 10.3389/fphar.2021.680172 (PMC8281221; doi:10.3389/fphar.2021.680172)
Supplement: Supplementary file 2 [file Table2.DOCX]

Supplementary material 2. Composition of Chinese patent medicines used in the included studies.

| Name of Chinese patent medicine | Source | Chinese name, pharmaceutical name, species, family, concentration | Quality control reported? (Y/N) |
| --- | --- | --- | --- |
| Xiaoyin granule | Shaanxi Kanghui Pharmaceutical Co., Ltd. Effective company | Dihuang, radix rehmanniae preparata, root of Rehmannia glutinosa (Gaertn.) DC., Orobanchaceae;  Mudanpi, cortex moutan, root bark of Paeonia suffruticosa Andr., Paeoniaceae;  Chishao, radix peoniae rubra, root of P. veitchii Lynch, Paeoniaceae;  Danggui, radix angelicae sinensis, root of Angelica sinensis (Oliv.) Diels, Apiaceae;  Kushen, radix sophorae flavescentis, root of Sophora flavescens Ait., Fabaceae;  Jinyinhua, flos lonicerae, flower of Lonicera japonica Thunb., Caprifoliaceae;  Xuanshen, radix scrophulariae, root of Scrophularia ningpoensis Hemsl., Scrophulariaceae;  Niubangzi, fructus arctii, ripe fruit of Arctium lappa L., Asteraceae;  Chantui, shell of Cryptotympana pustulata Fabricius;  Baixianpi, cortex dictamni, root bark of Dictamnus dasycarpus Turcz., Rutaceae;  Fangfeng, root of Saposhnikovia divaricate (Turcz. Ex Ledeb.) Schischk., Apiaceae;  Daqingye, folium isatidis, leaf of Isatis indigotica Fort., Brassicaceae;  Honghua, flos carthami tinctorii, flower of Carthamus tinctorius L., Asteraceae; | Y- Prepared according to Pharmacopoeia of the People’s Republic of China; Z20000019 |
| Biejiajian pill | Wuhan Zhonglian Pharmaceutical Group CO., Ltd. | Biejia, tergum of Trionyx sinensis Wiegmann, 3.6g;  Shegan, rhizome of Belamcanda chinensis (L.) DC., 0.9g;  Huangqin, radix scutellariae, root of Scutellaria baicalensis, Lamiaceae,0.9g;  Chaihu, radix bupleuri, root of Bupleurum chinensis DC., Apiaceae, 1.8g;  Ganjiang, rhizome zingiberis recens, rhizome of Zingiber officinale Roscoe, Zingiberaceae, 0.9g;  Dahuang, radix et rhizoma rhei, root and rhizome of Rheum officinale Baill., Polygonaceae, 0.9g;  Shaoyao, radix paeoniae alba, root of Paeonia lactiflora Pall., Paeoniaceae, 1.5g;  Guizhi, ramulus cinnamomi, burgeon of Cinnamomum cassia Presl, Lauraceae, 0.9g;  Tinglizi, semen lepidii seu desurainiae, mature seed of Lepidium apetalum Willd., Brassicaceae, 0.3g;  Shiwei, folium pyrrosiae, leaf of Pyrrosia lingua (Thunb.) Farw., Polypodiaceae, 0.9g;  Houpo, cortex magnoliae officinalis, bark of Magnolia officinalis Rehder & E.H.Wilson, Magnoliaceae, 0.9g;  Mudanpi, cortex moutan, root bark of Paeonia suffruticosa Andr., Paeoniaceae, 1.5g;  Qumai, herba dianthi, the above-ground part of Dianthus superbus L., Caryophyllaceae, 0.6g;  Banxia, pinelliae rhizoma, tuber of Pinellia ternate (Thunb.) Makino, Araceae, 0.3g;  Tubiechong, body of Eupolyphaga sinensis Walker, 1.5g;  Ejiao, solid glue of skin of Equus asinus L., 0.9g;  Fengfang, nest of Polistes olivaceous (DeGeer), 1.2g;  Taoren, semen persicae, mature seed of Prunus persica (L.) Batsch, Rosaceae, 0.6g;  Etc. | Y- Prepared according to Pharmacopoeia of the People’s Republic of China; Z42020772 |
| Yinxie capsule | Xinjiang Viatang Pharmaceutical Co., Ltd. | Tufuling, rhizoma smilacis glabrae, tuber of Smilax glabra Roxb., Smilacaceae;  Baqia, rhizome of Smilaz china L. | Y- Prepared according to Pharmacopoeia of the People’s Republic of China; Z20080093 |
| Total glucosides of paeony capsule | Ningbo Lihua Pharmaceutical CO., Ltd. | Total glucosides of paeony extracted from the root of Paeonia lactiflora Pall. | Y- Prepared according to Pharmacopoeia of the People’s Republic of China; H20055058 |
| Ruizao zhiyang capsule | Guizhou Tongjitang Pharmaceutical CO., Ltd. | Heshouwu, radix polygoni multiflori, root of Reynoutria multiflora (Thunb.) Moldenke, Polygonaceae;  Shengdihuang, radix rehmanniae preparata, root of Rehmannia glutinosa (Gaertn.) DC., Orobanchaceae;  Sangye, cortex mori radicis, leaf of Morus alba L., Moraceae;  Kushen, radix sophorae flavescentis, root of Sophora flavescens Aiton, Fabaceae;  Honghuoma, whole plant of Girardiana heterophylla Decne. | Y- Prepared according to Pharmacopoeia of the People’s Republic of China; Z20025030 |
| Kushenin tablet | Guangdong Yishu Pharmaceutical CO., Ltd. | Kushenin extracted from root of Sophora flavescens Ait. | Y- Prepared according to Pharmacopoeia of the People’s Republic of China; H20080335 |
| Dahuang zhechong pill | Beijing Tongrentang Co., Ltd. Tongrentang Pharmaceutical Factory | Shudahuang, radix et rhizoma rhei, root and rhizome of Rheum officinale Baill., Polygonaceae,  Tubiechong, body of Eupolyphaga sinensis Walker;  Shuizhi, body of Hirudo nipponia (Whitman);  Mengchong, body of Tabanux manda-rinus Schiner;  Qicao, larva of Holotrichia diomphalia Bates;  Ganqi, the dried product of plant resin of Toxicodendron vernicifluum (Stokes) F.A. Barkl.;  Taoren, semen persicae, mature seed of Prunus persica (L.) Batsch, Rosaceae;  Kuxingren, semen pruni armeniacae, mature seed of Prunus armeniaca L., Rosaceae;  Huangqin, radix scutellariae, root of Scutellaria baicalensis, Lamiaceae;  Dihuang, radix rehmanniae preparata, root of Rehmannia glutinosa (Gaertn.) DC., Orobanchaceae;  Baishao, radix paeoniae alba, root of Paeonia lactiflora Pall., Paeoniaceae;  Gancao, radix glycyrrhizae uralensis, root and rhizome of Glycyrrhiza uralensis Fisch. Ex DC., Fabaceae; | Y- Prepared according to Pharmacopoeia of the People’s Republic of China; Z11020002 |
| Yinxieling tablet | Preparation Department of Guangdong Provincial Hospital of Traditional Chinese Medicine | Chuanxiong, rhizoma ligustici chuanxiong, rhizome of Conioselinum anthriscoides ‘Chuanxiong’, Apiaceae;  Ezhu, radix curcumae, rhizome of Curcuma zedoaria (Christm.) Roscoe, Zingiberaceae;  Danggui, radix angelicae sinensis, root of Angelica sinensis (Oliv.) Diels, Apiaceae;  Chishao, radix peoniae rubra, root of P. veitchii Lynch, Paeoniaceae;  Shengdi, radix rehmanniae preparata, root of Rehmannia glutinosa (Gaertn.) DC., Orobanchaceae;  Zicao, radix arnebiae seu lithospermi, root of Lithospermum erythrorhizon Siebold & Zucc., Boraginaceae;  Wumei, fructus mume, fruit of Prunus mume (Siebold) Siebold & Zucc., Rosaceae;  Tufuling, rhizoma smilacis glabrae, tuber of Smilax glabra Roxb., Smilacaceae;  Etc. | Y- Prepared according to Pharmacopoeia of the People’s Republic of China; Hospital preparation |
| Wushe jiedu pill | Preparation Department of Dalian Hospital of Traditional Chinese Medicine | Wushaoshe, body that removed the internal organs of Zaocys dhumnades (Cantor);  Jiangchan, the dried body of larva of Bombyx mori infected with Beauveria bassiana (Bals.) Vaillant;  Shetui, skin of Elaphe taeniurus Cope;  Niubangzi, fructus arctii, ripe fruit of Arctium lappa L., Asteraceae;  Kushen, radix sophorae flavescentis, root of Sophora flavescens Ait., Fabaceae;  Danggui, radix angelicae sinensis, root of Angelica sinensis (Oliv.) Diels, Apiaceae;  Shengdi, radix rehmanniae preparata, root of Rehmannia glutinosa (Gaertn.) DC., Orobanchaceae;  Maidong, radix ophiopogonis, root of Ophiopogon japonicus (Thunb.) Ker Gawl., Asparagaceae;  Heshouwu, radix polygoni multiflori, root of Reynoutria multiflora (Thunb.) Moldenke, Polygonaceae;  Danshen, radix salvia miltiorrhizae, root and rhizome of Salvia miltiorrhiza Bunge, Lamiaceae;  Ezhu, radix curcumae, rhizome of Curcuma zedoaria (Christm.) Roscoe, Zingiberaceae;  Huangqi, radix astragali, root of Astragalus mongholicus Bunge, Fabaceae;  Gancao, radix glycyrrhizae uralensis, root and rhizome of Glycyrrhiza uralensis Fisch. Ex DC., Fabaceae; | Y- Prepared according to Pharmacopoeia of the People’s Republic of China; Hospital preparation |
| Keyin pill | NR | Tufuling, rhizoma smilacis glabrae, tuber of Smilax glabra Roxb., Smilacaceae;  Baixianpi, cortex dictamni, root bark of Dictamnus dasycarpus Turcz., Rutaceae;  Beidougen, asiatic moonseed rhizome, rhizome of Menispermum dauricum DC., Menispermaceae;  Quanshen, rhizoma bistortae, rhizome of Bistorta officinalis Delarbre, Polygonaceae; | NR |

NR, not reported.
